# UV Irradiation of Wood Surface: Bonding Properties

**DOI:** 10.3390/polym15112552

**Published:** 2023-05-31

**Authors:** Tomislav Gržan, Lucianna Grieco, Vjekoslav Živković, Goran Mihulja

**Affiliations:** 1 Faculty of Forestry and Wood Technology, University of Zagreb, 10000 Zagreb, Croatia; tgrzan@sumfak.unizg.hr (T.G.); vzivkovic@sumfak.unizg.hr (V.Ž.); 2 School of Agricultural, Forest, Food and Environmental Sciences, University of Basilicata, 85100 Potenza, Italy; lucianna.grieco@unibas.it

**Keywords:** UV light, UV irradiation, machining, surface modification, bonding properties

## Abstract

Various surface modification techniques have been developed to improve synthetic polymer surfaces’ wetting, adhesion, and printing by adding various functional (polar) groups. UV irradiation has been proposed as a suitable procedure to achieve adequate surface modifications of such polymers, which can be of further use to bond many compounds of interest. The activation of the surface, the favourable wetting properties, and the increased micro tensile strength of the substrate after short-term UV irradiation suggest that such pretreatment can also improve the bonding of the wood-glue system. Thus, this study aims to determine the feasibility of UV irradiation for pretreatment of wood surfaces before gluing and to determine the properties of wooden glued joints prepared in this way. UV irradiation was used to modify variously machined pieces of beech wood (*Fagus sylvatica* L.) before gluing. Six sets of samples were prepared for each machining process. Samples prepared in this manner were exposed to irradiation on a UV line. Each radiation level had a certain number of passes through the UV line, the more passes, the stronger the irradiation. Thus, the radiation levels were as follows: 1, 5, 10, 20, and 50 passes. The dose (energy delivered on the wood surface) in one pass was 2.36 J/cm^2^. A wetting angle test with glue, a compressive shear strength test of lap joints, and designation of main failure patterns were used to evaluate the properties of wooden glued joints. Wetting angle test was performed according to EN 828, while the compressive shear strength test samples were prepared and tested following the ISO 6238 standard. The tests were conducted using a polyvinyl acetate adhesive. The study found that UV irradiation before gluing improved the bonding properties of variously machined wood.

## 1. Introduction

Nowadays, almost every wooden product is glued at some point during production, and it is nearly impossible to imagine wood products, or rational uses of wood, without the bonding process. Due to its complex texture, wood is much more demanding to glue than other materials. Wood’s bonding strength refers to its ability to resist separation from its bonded components. A quality bonded joint will have high strength and durability only if the bonding process considers all the factors that may affect it. So, this process is highly complex and bonding performance is significantly affected by wood specie, adhesive type, surface preparation, and gluing technique [1,2,3].

Surface properties are important parameters that often determine wood’s potential uses [4,5,6]. Various surface preparations result in different surface properties (e.g., roughness, wettability, surface energy), which means that surfaces should be adequately treated to reduce their adverse impact. A good adhesive bond can be achieved when the liquid glue wets the wood surface well. Wetting can be improved by increasing the surface energy of the wood or by decreasing the surface tension of the glue state [7,8,9,10]. Using adhesives on inactivated wood substrates results in weak bonds, as the inactivation process reduces an adhesive’s ability to properly wet, flow, penetrate, and cure [1]. Thus, the surface must be altered or modified, better said activated, in some way. This can be accomplished through mechanical methods, chemical activation, or irradiation [1,11,12]. In recent years, several surface modification techniques have been developed to improve the wetting, adhesion, and printing of polymer surfaces by adding various functional (polar) groups [13,14,15,16]. Relevant techniques in polymer surface modification are wet chemical deposition, organosilane monolayer treatment, ionised gas treatments (e.g., plasma, corona discharge, flame treatment), machining processes (sanding or planing), and ultraviolet (UV) irradiation. In all techniques, functionalised polymer surfaces are produced, and/or the old inactive layer is removed, revealing a fresh surface of the polymer [16,17]. In contrast to other activation methods, UV irradiation can be applied in-line during manufacturing [18]. UV irradiation has been proposed as a suitable procedure to achieve adequate polymer surface modifications by generating active sites, which can be of further use to bond many compounds of interest [14]. As a result of UV light exposure, polymer surfaces produce reactive sites, which can become functional groups when exposed to gases or can be used to initiate UV-induced graft polymerisation [19].

Many researchers have applied the UV irradiation method to enhance adhesion at the interface of dissimilar materials, including polyethylene (PE)/polyetheretherketone (PEEK) [20], copper/epoxy, copper/graphene [21] rubber/polyurethane [22], polymethyl methacrylate (PMMA)/gold, PMMA/copper [23], silicon/gold [24], and carbon fibre reinforced PEEK/phenylene-sulfide [12]. Quan et al. reported that UV irradiation is a rapid, eco-friendly, and low-cost method for activating thermoplastic composite (TPC) surfaces, resulting in the excellent structural integrity of adhesive joints [12]. As a heterogeneous and porous biopolymer, wood presents more challenges to activate by UV light than synthetic polymer surfaces (smooth, homogeneous, non-porous, etc.) [16,18,25].

Bogner (1993) reports that electromagnetic radiation can increase the energy of wood’s surface layer. An increase in the amount of surface energy increases the wetting of the wood surface with glue, and thus the adhesion of the wood-glue system [26]. Wood absorbs light rapidly and intensely, especially in UV than in other areas of the spectrum [27]. The UV and visible radiation penetration depth into the wood is not great, so the changes are limited to the shallow surface layer. UV light is completely absorbed by a 75 to 80 μm-thick layer of wood’s surface [28,29]. Exposure of polysaccharides to UV irradiation is reported to enhance chemical reactions due to photooxidative degradation. The quantum energy associated with this process can break many chemical bonds present in wood components (carbon–carbon, carbon–oxygen, carbon–hydrogen bonds) [13,14]. Free radicals are the primary photochemical products generated by this homolytic process. It can cause depolymerisation and the formation of chromophoric groups, including carbonyls, carboxyls, quinones, peroxides, hydroperoxides, and conjugated double bonds, either with or without oxygen and water involvement. Secondary reactions, chain formation of free radicals, depend on the temperature and increased presence of oxygen and water [30,31,32,33]. The chemical components of wood (i.e., lignin, cellulose, hemicellulose, and extractives) are sensitive to UV radiation with a consequential deterioration effect [16,19]. Hon and Shiraishi (1991) asserted that lignin contributes 80–95% to the absorption coefficient of wood, carbohydrates 5–20%, and extractive substances about 2% [27]. Lignin is easily degraded in light shorter than 350 nm, so it is therefore very susceptible to degradation when exposed to the UV part of sunlight. Light with wavelengths longer than 350 nm does not degrade but causes bleaching and lightening of lignin. The initial and most prominent absorption occurs on chromophore groups within the phenolic molecular network of lignin, resulting in stronger decomposition than cellulose [34]. The presence of lignin protects cellulose from photodegradation to a certain extent because lignin exhibits pronounced absorption properties and a propensity for autoxidation [35].

On the other hand, the primary process in the photochemical degradation of cellulose is thought to be the breaking of its chains and this process is not dependent on the presence of oxygen [34,36]. It is believed that the organised regions of cellulose are impermeable to light due to their high degree of crystallinity, so depolymerisation occurs only in the light-accessible, amorphous regions [30]. However, the crystal surface is saturated with hydroxyl groups that are also capable of reacting with light [31]. According to the above, UV irradiation can clean the wood surface, causing pits to open, altering surface morphology, and to some extent, altering the chemical composition of the wood surface. The deterioration of connections between cell elements was related to the degradation of the middle lamella (S1 layer), which also confirmed the degradation of lignin [36,37]. Consequently, when hydrophobic lignin degrades, surface hydrophilicity increases due to abundant cellulose [18,38]. So, the effects of UV activation are most likely seen in surface morphology changes and surface free energy (wetting angle). UV irradiation of wood initiates surface oxidation (an increase in acid/base or polar components), resulting in functional group formation (carboxyl groups), which increases surface free energy, wettability (decreased contact angles), surface roughness, and adhesion [39,40,41,42]. The destruction of pits allows coatings and adhesives to penetrate deeper into wood surfaces, enhancing mechanical anchoring [18]. UV irradiation also increases the strength of the surface layer of wet wood [33,35].

The activation of the surface, the favourable wetting properties, and the increased micro-tensile strength of the substrate after short-term UV irradiation suggest that such pretreatment can also improve the bonding of the wood-glue system. Thus, this study aims to determine the feasibility of UV radiation for the pretreatment of wood surfaces before gluing and to determine the properties of wooden glued joints prepared in this way. Therefore, UV irradiation was used to activate face-milled, planed, and sanded surfaces of beech wood (*Fagus sylvatica* L.) before gluing.

## 2. Materials and Methods

### 2.1. Wood

The study was performed with slightly steamed beech wood (*Fagus sylvatica*), obtained from a local supplier. Kiln-dried boards of approximately 1000 × 150 × 30 mm^3^ (L × R × T) were formatted into 1000 × 130 × 13 mm^3^ (L × R × T) boards with a circular saw followed by face milling to ensure the flatness and uniformness for further mechanical processing. Six high-quality boards were chosen: without bio-damages, knots, or other inhomogeneities; with radial texture—the angle between the surface and annual rings was 60°–90°. This is particularly important since it has been shown that a flat growth ring angle is related to poor bonding performance [43,44]. Prior to other technological operations, boards were conditioned at a temperature of 23 ± 2 °C and a relative air humidity of 50 ± 5%, achieving an equilibrium moisture content of 9 ± 2%.

### 2.2. Wood Surface Machining

Wood surfaces were prepared first mechanically and then by UV irradiation. The surfaces were mechanically prepared through three surface machining methods: planing, sanding, and face milling. Face milling was performed on Homag Venture 316M CNC machining centre (Zagreb, Croatia), while planing was performed on the combined planer and thicknesser Rojek, MSP 415. Planing cutter head had four sharpened knives, and the face milling cutter head had three newly installed insertion knives (cutting edges). Sanding was carried out with a manual oscillating sander-belt Makita provided with a new belt of grit P120. Additional machine characteristics and process parameters are provided in Table 1. Therefore, 1000 × 130 × 13 mm^3^ (L × R × T) boards were surface machined differently and then sized to produce three sets of 24 boards measuring 200 × 130 × 10 mm^3^ (L × R × T).

**Table 1:** Surfacing machines and process parameters.

Surface Method	Sanding	Planing	Face Milling
Surfacing Machine	Manual oscillating sander Makita	Combined planer and thicknesser Rojek, MSP 415	CNC machining centre Homag Venture 316M
Characterisation of Knives/Sanding Belt	P120	VHW	HW, insertion knives with four-sided blades and rake angle 35 *
Tool Diameter (mm)	n/a	95	80
Number of Cutting Edges, *z*	n/a	4	3
Cutting Speed, v_c_ (m/s)	n/a	20.89	83.78
Feed Speed, v_f_ (m/min)	5	5	5
Feed, f_z_ (mm)	n/a	0.3	0.08
Cutting Depth (mm)	0.5	0.8	0.8
Rake Angle (°)	n/a	25	−10 *
Clearance Angle (°)	n/a	27	47 *
Cutter (rpm)	n/a	4200	20,000

**Figure 1:**
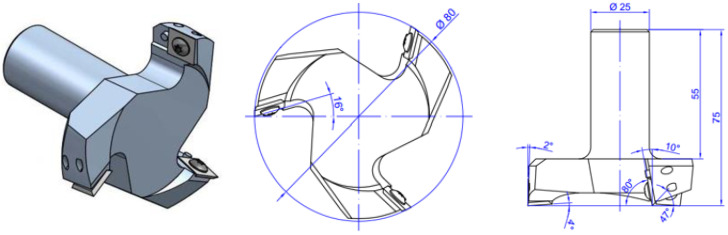
Face milling tool shown in isometry and orthogonal projection.

* As the blade is highly specific, a detailed view of the tool is presented in Figure 1.

### 2.3. UV Irradiation

After mechanical preparation, surfaces were additionally modified by UV irradiation. For each method of surface treatment, six sets were prepared, five sets for different levels (more in Section 2.3.2.) of irradiation and one set as a control. There were four boards in each set measuring 200 × 130 × 10 mm^3^ (L × R × T). Furthermore, the boards were cut into two parts before UV irradiation. One part was used for measuring adhesive wetting angles (27 × 125 × 10 mm^3^, L × R × T), and the other one for preparing shear test specimens (170 × 130 × 10 mm^3^, L × R × T). Once the boards had been mechanically treated and cut, they were placed in a darkened plastic box without the presence of light until they were exposed to UV radiation. The box was stored in the standard laboratory environment. UV irradiation was performed within 12 h of mechanical treatment.

#### 2.3.1. Work Principles of the UV Line

**Figure 2:**
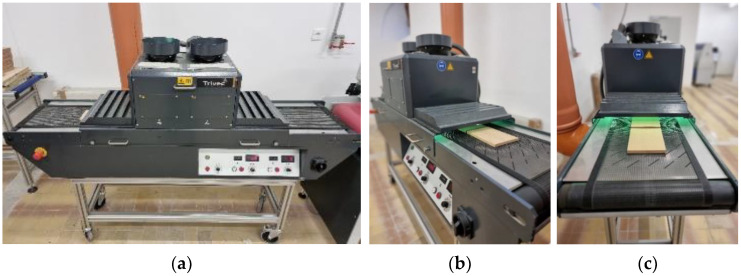
UV irradiation process: UV line Trivec (**a**), input (**b**), and output (**c**) of specimens.

Each radiation level had a certain number of passes through the UV line, the more passes, the stronger the radiation was. Irradiation of the surface of the samples was carried out on a device for UV drying and hardening of varnishes from brand Trivec (Figure 2). The device consists of horizontal, powered input and output tables, electric folding panels (integrated into the lower part of the case), UV exposure units radiation, exhaust fan, and transport engine. The device works on the principle of passing the boards under UV lamps where it changes the distance from the lamp, the belt speed (%), and the intensity of the lamp irradiance (W/cm) to achieve a certain dose of radiation (J/cm^2^). The device contains mercury (Hg) and gallium (Ga) lamps.

#### 2.3.2. Determination of the Irradiation Dose

**Figure 3:**
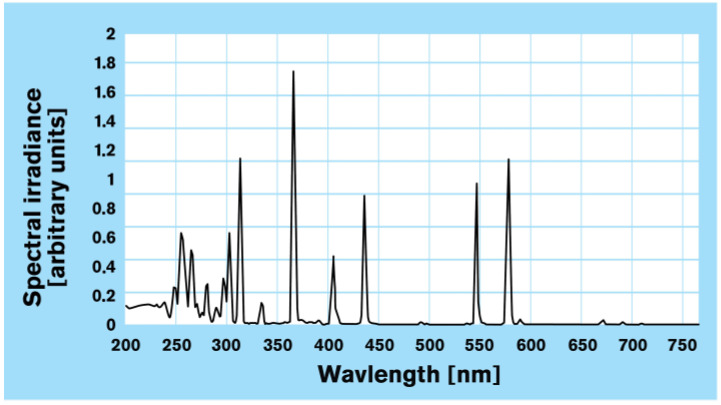
UV emission spectrum of the medium-pressure Hg-lamp (Heraeus) used (Heraeus brochure).

During irradiation, the samples were passed under the UV lamp which was positioned 130 mm from the surface of the specimen, at the lowest belt speed, and at the highest intensity. For this research, a medium-pressure Hg-lamp (Heraeus brand) with a UV emission spectrum according to Figure 3, was used. Medium-pressure UV lamps have a broadband emission in the UV and visible light spectral range, therefore spectral lines from 200 nm to 600 nm have high radiation output in the UVC spectrum. Their strong UV radiation flux results in high penetration depth and efficient disinfection. Their high UV intensity also makes them particularly suitable for photochemical processes (Heraeus brochure). Further characteristics of the lamp are shown in Table 2.

Due to the fact that the purpose of this work is to demonstrate the general ability of UV light to activate different wooden surfaces before gluing, working at a constant distance makes more sense, as it minimises measurement variation. One of the possible optimisation steps after confirmation of the activation of wood surfaces by UV light is adjusting the distance between the UV lamp and the wood surface. Under previously mentioned conditions, it was measured with a radiometer that the irradiation dose during one pass of the specimen under the lamp is 2.36 J/cm^2^. Irradiation levels were performed in 1, 5, 10, and 50 passes. The total intensity of radiation was determined by multiplying the radiation dose during one pass (2.36 J/cm^2^) and the number of passes of the sample under the lamp (Table 3).

### 2.4. Adhesive and Wood Bonding

The adhesive used was polyvinyl acetate adhesive (PVAc, Kleiberit 300.0, Weingarten, Germany), whose properties are listed in Table 4. As indicated in the literature, polyvinyl acetate is a thermoplastic adhesive and belongs to the prepolymerized adhesives category [45].

The bonding material was 72 pieces of flat, differently machined, and UV-irradiated boards 170 mm × 130 mm × 10 mm (L × R × T). The adhesive was applied one-sided with a spread of 200 g/m^2^ (A = 0.0221 m^2^; spread = 4.42 g per joint), manually, using a metal-toothed spatula. The bonding process was performed in the press for 60 min at a pressure of 1 N/cm^2^. The bonding operations were all performed within the specifications of the adhesive manufacturers. Bonding was performed immediately after UV irradiation, i.e., within 12 h after machining. Upon the hardening of the adhesive, two glued boards (170 mm × 130 mm × 20 mm) were obtained for each combination of mechanical treatment and UV irradiation level, from which 32 shear-test specimens were made. Therefore, from each glued board, 16 lap-shear specimens 35 × 25 × 20 mm^3^ (L × R × T) were prepared according to the instructions of ISO 6238 standard [46] (more in Section 2.6). All steps of the bonding process and seven-day specimen conditioning before shear testing were carried out under standard laboratory conditions.

### 2.5. Wetting Angle

**Figure 4:**
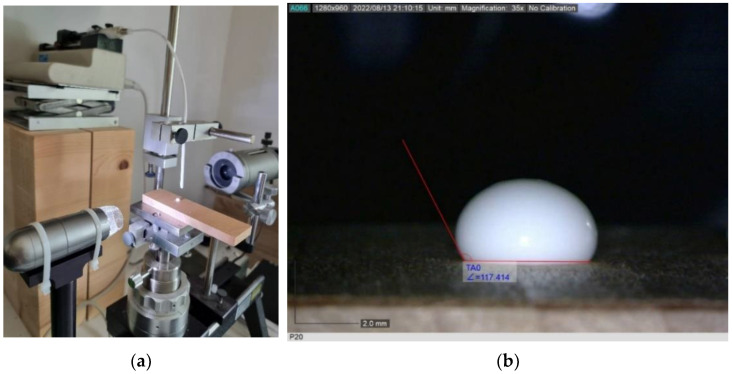
Device for measuring the wetting angles—goniometer OCA20, Data Physics (**a**) and wetting angle measurement and evaluation in the Dinolite 2.0 capture program (**b**).

On the radial textured specimens, dimensions of 30 × 130 × 10 mm^3^ (L × R × T), the adhesive (PVAc) wetting angle was measured using the sessile drop method with instruction of EN 828 standard [47]. The device for measuring the wetting angle consisted of a Dinolite 2.0 digital camera, lighting fixture, a specimen and needle stand, and a control device to adjust the volume and dosage of drops (Figure 4a). A total of 24 specimens were tested for each surface treatment with different levels of irradiation: therefore, 4 specimens for each of the 6 levels of irradiation (5 levels and control). Five drops were recorded on each specimen, and the volume of each drop was 4 μL. Thus, a total of 20 measurements were performed on each type of prepared sample. The test was performed under standard laboratory conditions immediately after UV irradiation, i.e., within 12 h after machining. Each adhesive drop was recorded for about a minute, and it was found that the droplet stopped changing and assumed its final shape after about 10 s. Thus, the wetting angle between a liquid drop and a wood surface was determined to be 10 s after applying the adhesive drop. The recordings were displayed, the final figures of drops were captured, and the wetting angles were evaluated and measured in the Dinolite 2.0 capture program (AnMo Electronics Corporation, New Taipei City, Taiwan) (Figure 4b).

### 2.6. Bonding Quality

Bonding quality was expressed through the shear strength test and designation of main failure patterns. The bonded beech wood adhesion strength test—the compressive shear strength of lap joints was performed and evaluated by the standard ISO 6238 [46].

**Figure 5:**
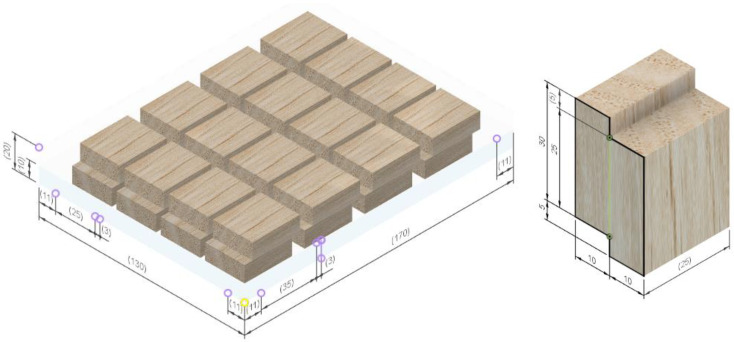
Compression shear strength test specimen according to ISO 6238: Layout of the specimen (**right**) and schematic representation of its production (**left**). Shear loaded bonding surface is shown in green (modified according to [46]).

The shear test was performed using a 100 kN universal mechanical testing machine Shimadzu (Kyoto, Japan). A total of 572 specimens were tested and evaluated for three surface treatments with six levels of irradiation: therefore, 32 specimens for each combination. The specimens were tested at a constant testing speed until the separation of the substrates in the force-controlled mode indicated by the standards. Using the maximum load observed at the breakpoint (F_max_ in Newtons) and the bonding surface of the sample (mm^2^) (a is glue face width, and b is glue face length—shown in Figure 5), shear strength was calculated according to the following equation:

(1) $$S=\frac{F_{\max}}{a\times b}\;,\;\left[\frac{N}{{\mathrm{mm}}^{2}}\right].$$

After the shear test, the specimen’s fracture surfaces were evaluated for the following main failure patterns: wood failure percentage (WFP), glue failure percentage (GFP), interface failure percentage (IFP), and adhesion failure percentage (AFP). Both sides of the failure surfaces of the specimen were examined visually, using a light source to illuminate the bonding surface at a specific angle, as recommended by the standard [46]. The failure percentage was evaluated at an approximation of 5%. To facilitate identification and evaluation, surfaces were contrasted with Lugol’s indicator solution (1% iodine) [46].

### 2.7. Statistical Analysis

Statistical analysis and data visualisation was performed using the R (ver. 4.2.0) and R Studio software with the help of ‘ggstatsplot’ [48] and ‘ggplot2’ [49] packages. Analysis of variance (ANOVA) was used to evaluate the variation of adhesive wetting angles and shear strength of wood joints grouped by type of surface machining. The source of variation was the UV irradiation dose. The Shapiro–Wilk test confirmed the normality of the data, and Levene’s test checked the homogeneity of the variances. When the assumption of homogeneity was met, a one-way ANOVA test was used; if not, Welch’s ANOVA test was used. A *p*-value, ω^2^, and a 95% confidence interval were used to estimate the effect size in omnibus tests. Effect size (ω^2^) of omnibus test was interpretated as per Fields’s (2013) conventions: ω^2^ < 0.01—very small, 0.01 ≤ ω^2^ < 0.06—small, 0.06 ≤ ω^2^ < 0.14—medium, ≥0.14—large [50]. An appropriate pairwise comparison (post hoc) test was performed if the omnibus test proved statistical significance. Thus, the Student’s pairwise test (equal variances) was used after the one-way ANOVA, while the Games Howell pairwise test (non-equal variances) was used after Welch’s ANOVA. Correlation and strength of relationships between the two variables were indicated with the Pearson correlation coefficient. The effect sizes of the pairwise test and Pearson correlations coefficient were estimated using holm-adjusted *p*-values. Descriptive statistics were also used to display the results: arithmetic means (red points), standard deviation, median, and 25th and 75th percentiles. Graphic displays were made using box whiskers and bar plots. The results are shown with a 95% family-wise confidence level.

## 3. Results and Discussion

### 3.1. Wetting Angles

**Figure 6:**
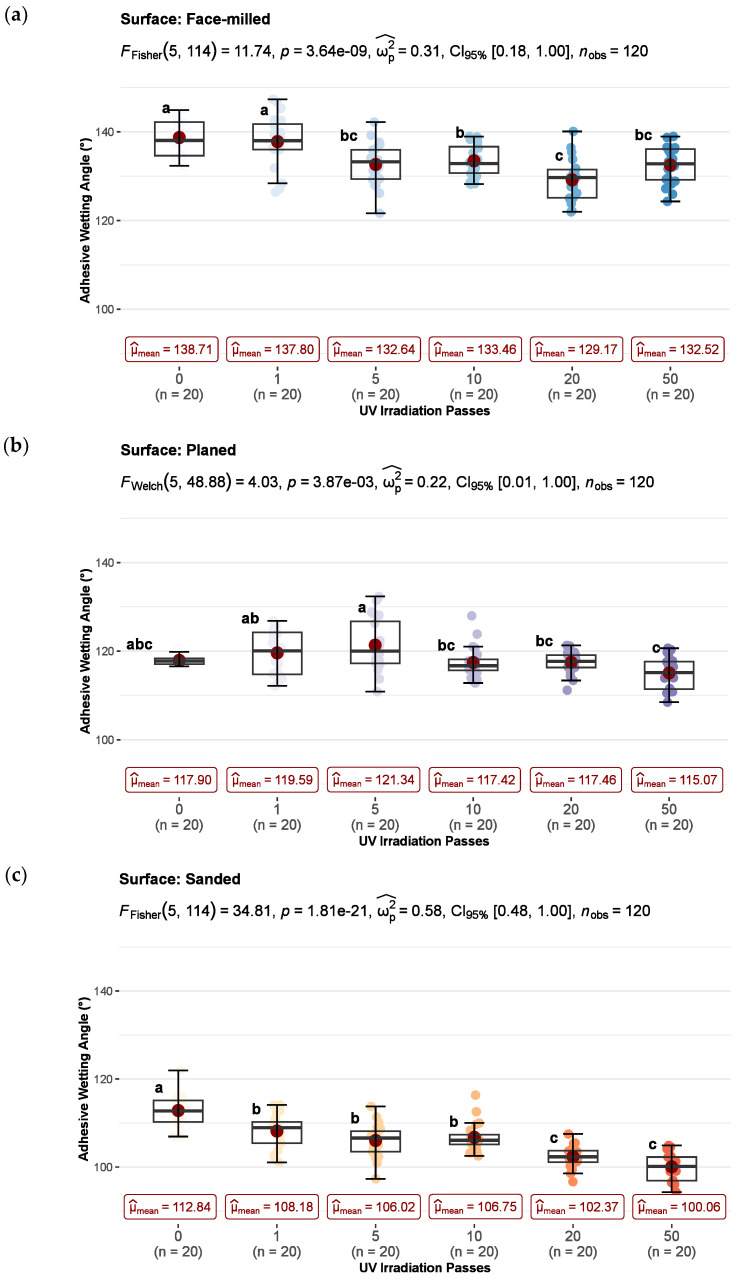
The effect of UV-irradiation before gluing on the adhesive wetting angles of face-milled (**a**), planed (**b**), and sanded (**c**) surface: whiskers show 5th–95th percentile, red point in box displays arithmetic mean value, and horizontal line shows median (means within the UV irradiation level followed by the same letter are not significantly different at 5% level of significance).

The UV irradiation influenced the wetting of the differently machined beech wood surfaces, which was determined by adhesive wetting angle measurements. The results of the measurement of adhesive wetting angle on untreated and UV-irradiated wood surfaces are shown in Figure 6. One-way ANOVA was performed for each type of surface machining to compare the influence of UV irradiation on the adhesive wetting angle of differently treated surfaces. There was a statistically significant difference in mean wetting angle values between at least two levels of UV irradiation at the *p* < 0.05 for face-milled (F_F_ (5, 114) = 11.74, *p* = 0.000), planed (F_W_ (5, 48.9) = 4.03, *p* = 0.004), and sanded (F_F_ (5, 114) = 34.81, *p* = 0.000) surfaces. Additionally, the effect sizes indicate that the effect of UV irradiation in all three methods of surface machining, planing (ω_p_^2^ = 0.22), sanding (ω_p_^2^ = 0.58), and face milling (ω_p_^2^ = 0.31) were large (ω_p_^2^ ≥ 0.14—large).

**Figure 7:**
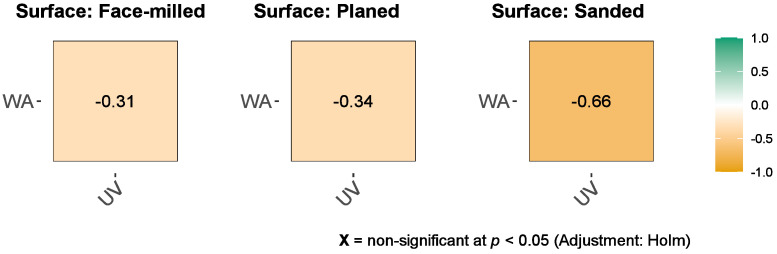
The bivariate correlation between UV irradiation (UV) and adhesive wetting angle (WA) for the face-milled, planed, and sanded joints are represented using a color-coding scheme. Dark green is associated with Pearson Correlation Coefficient, $r_{\mathrm{Pearson}}$, equal to 1 and dark orange is associated to $r_{\mathrm{Pearson}}$ = −1. The Pearson correlation coefficients are indicated on plot.

Also, based on the Pearson correlation test, all mechanical treatments tended to decrease adhesive wetting angles (e.g., better wettability) with increasing UV irradiation dose. There was a weak negative correlation between adhesive wetting angle and UV irradiation dose in face milling (r_Pearson_ = −0.31) and planing (r_Pearson_ = −0.34), but a strong negative correlation in sanding (r_Pearson_ = −0.66) (Figure 7).

Furthermore, multiple comparisons test found that the mean values of adhesive wetting angles were significantly different between some irradiation levels compared with control. This difference does not have the same trend for all surface preparations.

Wetting angles of the face-milled surfaces indicate that control and short-term irradiated samples (1 pass) belong to group a, whereas the other samples belong to groups b, bc, and c. The adhesive wetting angle was significantly lower for all radiation doses except 1 pass (2.36 J/cm^2^) compared to the control. Although the maximum wetting was achieved after 20 passes (45.20 J/cm^2^), a statistically significant improvement in wetting already occurred after 5 passes, and after that, there was no statistically significant difference compared to other irradiation doses. There was no significant difference between 20 (c), 5 (bc), and 50 passes (bc), but only between 10 (b) and 20 passes (c) (Figure 6a). In the case of planed surfaces, there was no statistically significant difference between the control sample (abc) and irradiated samples (a, ab, b, or c) in adhesive wetting angle. If the wetting angle is compared only between doses of irradiation, then there were statistically significant differences in some cases. Irradiation dose of 10 (bc), 20 (bc), and 50 (c) passes had a significantly lower adhesive wetting angle than 5 passes (a). In comparison, 50 passes (c) had a lower wetting angle than 1 (ab) and 5 passes (a) (Figure 6b). The effect of UV irradiation on the wettability of planed surfaces has not been statistically proven.

The statistically significant wetting improvement of sanded surfaces was achieved after one pass (b) of UV irradiation. A further significant improvement occurred after 20 passes (c) (Figure 6c). In general, sanded surfaces showed the lowest wetting angles in all investigated samples, followed by planed and face-milled surfaces. This is expected because sanded surfaces show good wettability and high process roughness [51]. A thin layer of wood flour combined with a high level of fibrillation created by sanding provides the best conditions for water spreading [52]. Further, more hydrophilic active groups (hydroxyl groups) are exposed on the surface, reducing the angle of contact between water-based glues or coatings and the wood [53,54].

### 3.2. Bonding Quality

**Figure 8:**
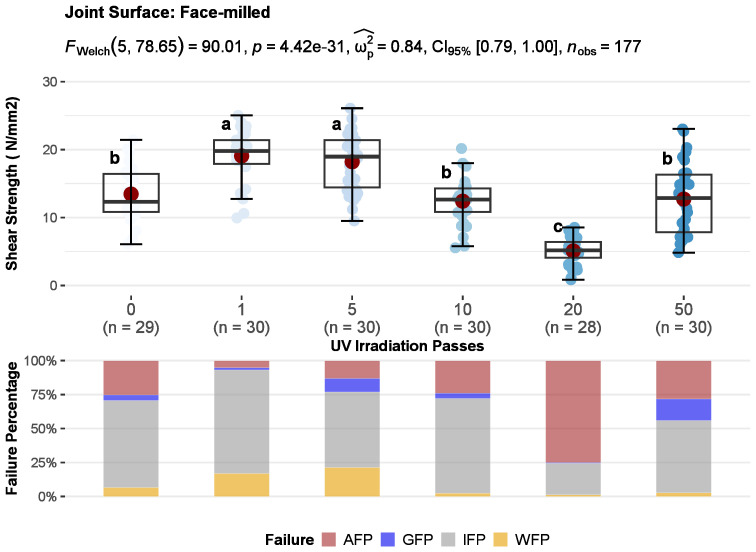
The effect of UV irradiation before gluing on the compressive shear strength (**top**) and average percentages of main failure patterns (**bottom**) of face-milled joints: whiskers show 5th–95th percentile, red point in box displays arithmetic mean value, and the horizontal line shows median (means within the UV irradiation level followed by the same letter are not significantly different at 5% level of significance).

**Figure 9:**
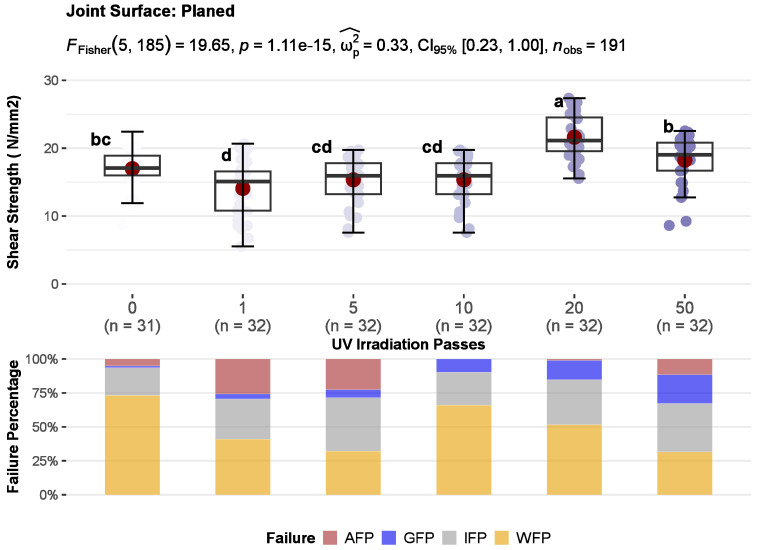
The effect of UV irradiation before gluing on the compressive shear strength (**top**) and average percentages of main failure patterns (**bottom**) of planed joints: whiskers show 5th–95th percentile, red point in box displays arithmetic mean value, and horizontal line shows median (means within the UV irradiation level followed by the same letter are not significantly different at 5% level of significance).

**Figure 10:**
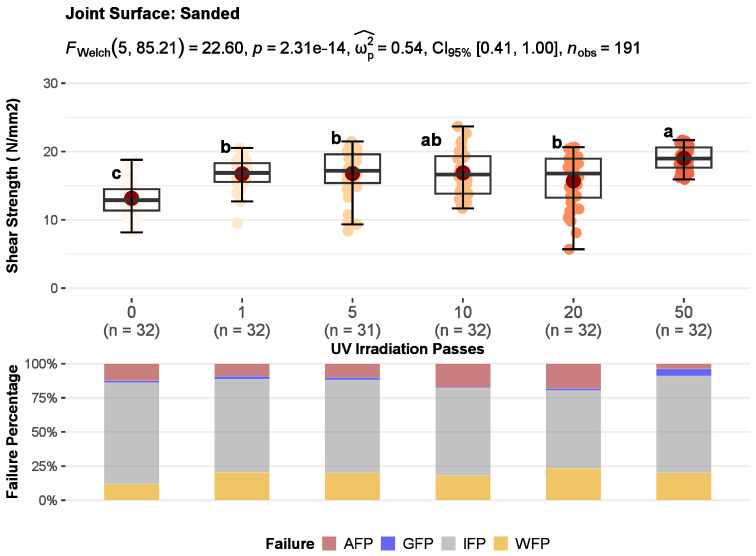
The effect of UV irradiation before gluing on the compressive shear strength (**top**) and average percentages of main failure patterns (**bottom**) of sanded joints: whiskers show 5th–95th percentile, red point in box displays arithmetic mean value, and the horizontal line shows median (means within the UV irradiation level followed by the same letter are not significantly different at 5% level of significance).

The results of the joints shear strength for untreated and UV-irradiated wood surfaces divided according to surface machining are shown in Figure 8, Figure 9 and Figure 10. One-way ANOVA was performed for each type of surface machining to compare the effect of UV irradiation on joint shear strength results. There was a statistically significant difference in mean shear strength values between at least two levels of UV irradiation at the *p* < 0.05 for face-milled (F_w_ (5, 78.7) = 90.01, *p* = 0.000), planed (F_F_ (5, 185) = 19.65, *p* = 0.000), and sanded (F_W_ (5, 85.2) = 22.60, *p* = 0.000) surfaces. As well, the effect sizes indicate that the effect of UV irradiation in all three methods of surface machining, planing (ω_p_^2^ = 0.33), sanding (ω_p_^2^ = 0.54), and face milling (ω_p_^2^ = 0.33) were large (ω_p_^2^ ≥ 0.14—large). Furthermore, multiple comparisons test found that the mean values of shear strength was significantly different between some irradiation levels compared with control. It indicates that increases in shear strength depend on the UV irradiation dose and the surface machining used.

**Figure 11:**
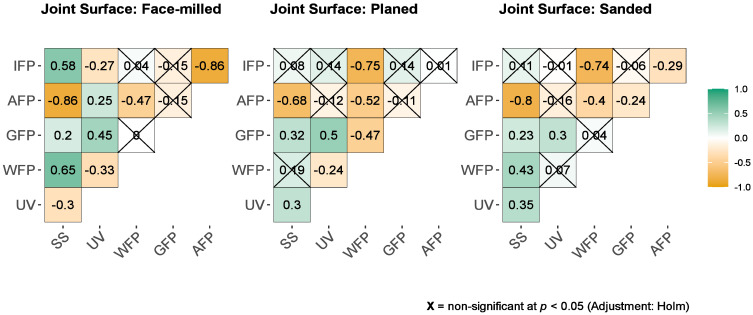
The bivariate correlations between UV irradiation (UV), shear strength (SS), wood (WFP), glue (GFP), interface (IFP) and adhesion (AFP) failure percentage for the face-milled, planed, and sanded joints are indicated using a color-coding scheme. Dark green is associated with Pearson Correlation Coefficient, $r_{\mathrm{Pearson}}$, equal to 1 and dark orange is associated to $r_{\mathrm{Pearson}}$ = −1. The Pearson correlation coefficients are represented on plot.

Also, the correlation coefficients between UV radiation dose and shear strength showed a weak negative correlation for face-milled joints ($r_{\mathrm{Pearson}}$ = −0.3), while sanded ($r_{\mathrm{Pearson}}$ = 0.35) and planed ($r_{\mathrm{Pearson}}$ = 0.3) joints showed a weak positive correlation (Figure 11). Generally, when increasing the dose of UV irradiation, the strength of face-milled joints decreases, and the strength of sanded and planed joints increases. However, with increasing the UV irradiation dose, the trend of shear strength did not follow the same pattern for each mechanical surface treatment. Therefore, a detailed analysis is given in the following text.

Joints with face-milled and sanded surfaces showed a statistically significant increase in shear strength after 1 pass of UV irradiation (2.36 J/cm^2^). That increase of 27% in sanded joints was approximately constant up to the dose of 20 passes (group b) and then at the dose of 50 passes (group a), it additionally increased by 45% regarding control (Figure 10). Contrarily, in face-milled joints, the increase of 42% was constant up to 5 passes and then sharply decreased with increasing UV irradiation (Figure 8). So, doses of one and five passes had statistically significant higher shear strength than control sample and other sample doses. The control, doses of 10 and 50 passes, belong to group b and there was no statistically significant difference in shear strength between them. The dose of 20 passes had statistically significant lower shear strength than other samples. At the dose of 10 passes, the shear strength is equal to the control, then decreases sharply and shows only 40% of the control shear strength at the 20 passes (45.2 J/cm^2^). Interestingly, with higher doses of 20 passes, the shear strength does not decrease further, at 50 passes it shows the same values as the control. In the case of joints with planed surfaces, a statistically significant improvement of 22% in shear strength was determined after 20 passes of UV irradiation (45.2 J/cm^2^) (Figure 9). However, joints with planed surfaces had a decrease in shear strength by 17% after 1 pass of UV irradiation.

The measured strength in some cases may or may not be a suitable criterion for determining bonding quality. It is very important to support the shear strength results with the type of failure in the joint. Surface fracture analysis and estimation of main failure patterns give more information about joint bonding quality than pure shear strength. Wood failure is common with quality glued joints and indicates that the strength of the joint (wood impregnated with glue) is greater than the cohesive strength of the wooden substrate [1]. However, with such failure, the actual shear strength of the wood-glue composite layer remains unknown. It is assumed to be equal to or greater than the one obtained. Therefore, the interface failure indicates the real shear strength of the wooden joint. The most preferred types of failure are cohesive wood failure and interface failure. Adhesion and glue failure are unfavourable patterns of surface fracture, although adhesive failure often shows high shear strengths due to the high cohesive strength of the adhesive. Therefore, stacked bar plots in Figure 8, Figure 9 and Figure 10 show the average percentages of main failure patterns by the radiation doses for each surface machining. In Figure 11, the main failure patterns of the joints are contrasted on all fracture surfaces of the samples.

**Figure 12:**
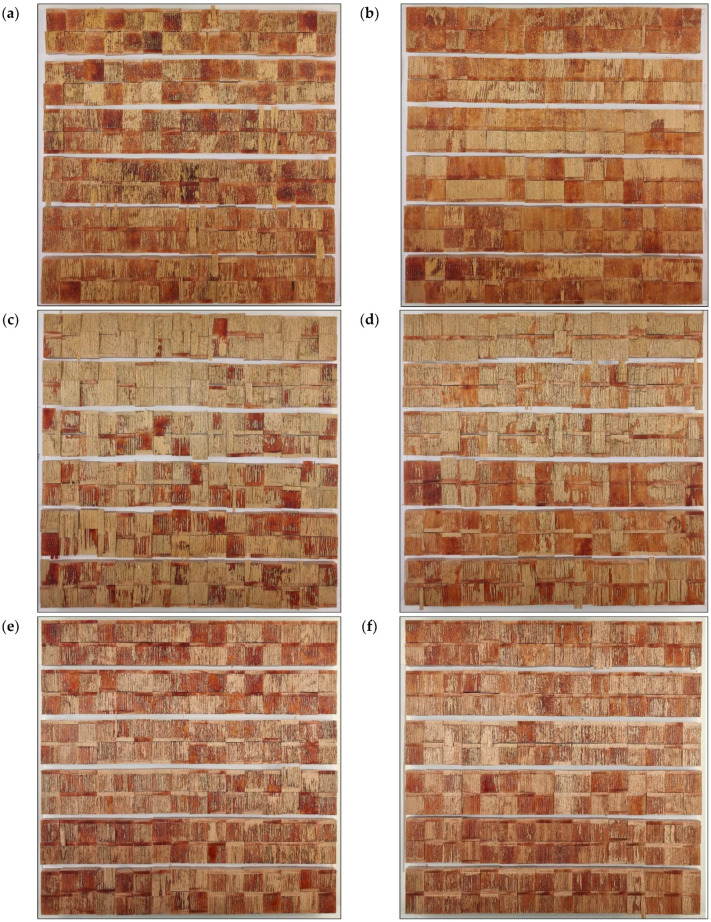
Main failure patterns of joints with: face-milled (**a**,**b**), planed (**c**,**d**), and sanded (**e**,**f**) surfaces before (**a**,**c**,**e**) 0, 1, 5 passes, and (**b**,**d**,**f**) 10, 20, 50 passes of UV irradiation. Every two rows of specimens represent a different dose of UV irradiation.

It is evident that with all doses of irradiation, except for 20 passes, the failure was primarily in the interface layer of face-milled joints (Figure 8, Figure 11 and Figure 12a–b). This confirms the joint’s shear strength since the fracture was initiated correctly and shows the shear strength of the glue-impregnated wood layer. As well, it is obvious that the change in shear strength depended on the percentage of adhesion failure. The adhesion failure was dominant at the dose of 20 passes, hence the low shear strength. So, on one surface of the joints, pure glue layer was visible (red to red-brown surface), and on the other, no traces of glue were visible (the surface is the color of wood); there were no wood fibers on the glue layer or traces of individual surface fibers were barely noticeable (Figure 6b, middle two rows). Based on the Pearson correlation test (Figure 11), the decrease in the shear strength of the joint is strongly related to the increase in adhesion failure in the face-milled ($r_{\mathrm{Pearson}}$ = −0.86), planed ($r_{\mathrm{Pearson}}$ = −0.68), and sanded ($r_{\mathrm{Pearson}}$ = −0.80) joint surface. However, a weak positive correlation between adhesion failure percentage and UV irradiation dose was only observed in the face-milled joints ($r_{\mathrm{Pearson}}$ = 0.25).

In the case of planed joints, the failure was primarily in wood, but in some doses, it was equally in the wood and interface layer. By increasing the dose of UV irradiation, the wood failure slightly decreased ($r_{\mathrm{Pearson}}$ = −0.24), but glue failure increased ($r_{\mathrm{Pearson}}$ = 0.5), and it became more pronounced after a dose of 10 passes (Figure 9, Figure 11 and Figure 12c,d). In lower doses of irradiation (1–5 passes) adhesion failure was present. Compared to the control, at doses of 10 and 20 passes, no adhesion failure occurs. Therefore, the satisfying failure model and the significant increase in strength make the dose of 20 passes the most favourable for the UV modification of planed surfaces before gluing. In planing, high pressure is exerted on the wood cells, whereby they are crushed, and the surface becomes plasticised [1]. Therefore, a higher irradiation dose was required to activate such a compressed and plasticised surface.

The failure of sanded joints dominantly occurred in the interface layer (Figure 12e–f). As shown in Figure 10 and Figure 11, increasing UV irradiation did not result in significant changes in failure patterns.

Therefore, after only one pass of UV irradiation (2.36 J/cm^2^), the shear strength of joints with face-milled and sanded surfaces improved statistically significantly, but for joints with planed surfaces, it improved after 20 passes (45.2 J/cm^2^). Several factors may contribute to the difference in shear strength between surface machining methods including surface roughness, fibrillation, and cell damage under the surface [55]. The results could be explained by the fact that sanding and face milling commonly cause cell wall fibrillation (surface fibrillation) [56], whereas planing crushes rather than cuts the cells, which results in the plasticisation of the surface. As a result of fibrillation and a proper longitudinal cell cut, cells are exposed on the surface, allowing UV light to intensively reach the pits and middle lamella. In contrast, light cannot reach the middle lamella as intensely on plasticised surfaces, where cells are crushed rather than cut. A direct result of UV light’s effect on wood cells exposed in this manner is the degradation of lignin. Additionally, the deterioration of connections between cellular elements is associated with the degradation of the middle lamella (layer S1), which also confirms the degradation of lignin [36,37]. When hydrophobic lignin degrades, surface hydrophilicity is increased because cellulose becomes more abundant on the wood surface [18]. A sharp increase in shear strength was therefore the result of the glue’s better mechanical and chemical adhesion. More surface area is available for mechanical anchorage with an adhesive, potentially contributing to improved adhesion strength. Moreover, UV light also causes pits to open, allowing coatings and adhesives to penetrate deeper into the wood, enhancing mechanical anchoring [16,18,57]. Beechwood is a diffuse-porous hardwood specie with small, uniformly distributed vessel element cells (pores) in each growth ring, making their anatomical structure more predictable compared to ring-porous hardwoods. As a result, this wood’s structure was not having an impact on the process of UV irradiation, gluing, and fracture initiation, even though it is an anisotropic biopolymer.

In addition, machining results in considerable deformation of the wood surface, resulting in a weak boundary layer. A chemically weak boundary layer on the molecular level and/or a mechanically weak boundary layer on the macro level may prevent adhesives and coatings from penetrating and anchoring to intact wood [16,57]. So, according to the conclusions stated earlier, UV light successfully activates and modifies the weak boundary layer by cleaning the wood surface, altering surface morphology, and to some extent, altering the chemical composition of the wood surface.

However, after the sharp increase in shear strength of face-milled and sanded joints with short-term intense UV radiation, the shear strength trend did not follow the same pattern. In both cases, the shear strength was initially relatively constant, and then, at higher doses of irradiation, it increased in the sanded joints, while it decreased in the face-milled joints. The values of the wetting angles of face-milled surfaces do not correlate directly with the shear strength, but it is one of the components that should be considered. The surface energy of the sanded surface has a greater share in the total adhesion than that of face-milled surfaces, which is also visible at lower wetting angles (Figure 6). UV exposure caused a slight decrease in the contact angle (increase in wettability) [18,57].

It has been suggested by Mihulja et al. (1999) that the decrease in shear strength of face-milled joints is the result of excessive drying of the surface by ultraviolet radiation, as well as too fast drying of the glue applied to the surface [42]. Besides the weak relationship between wetting and shear strength at high doses of radiation, high proportion of adhesion failure can also support this conclusion (Figure 8 and Figure 11b). When the glue is applied to such a surface, it dries rapidly and then cannot adhere to another wood piece adhesively, especially when it is one-side applied. For example, extending the adhesive’s open time in such cases would be desirable. The above is not the case with sanding and planing because the wetting angles decreased (wetting increased), and the shear strength increased when the radiation dose was increased. Moreover, the surface morphology after machining prevented undesirable glue and surface factors. It is known that a layer of crushed cells characterises sanded wood surface and subsurface, lumens clogged by fine dust, scratches, and packets of microfibrils torn out from cell walls [58,59,60]. Furthermore, sanding produces smooth, homogeneous surfaces for applying glues and defect-free surfaces for absorption of coatings [58]. Conversely, the face milling process enhanced the penetration of adhesives, especially for hardwood species, and produced significantly greater tensile shear strength than planing [55,60].

## 4. Conclusions

In this research, it was found that UV light, with its intense effect on the wood surface, improves the bonding properties (shear strength, wetting angle) of variously machined beech wood (*Fagus sylvatica* L.). The reason for this is that UV irradiation probably cleans wood surfaces, alters surface morphology, and to some extent, modifies chemical composition, thus activating and modifying weak boundary layers. Moreover, the dose of UV radiation affected the bonding properties differently depending on the surface machining.

The adhesive wetting angle decreased (increase in the energy of the surface layer of wood) while shear strength increased significantly for all tested surfaces with processing time and absorbed UV light. In joints with face-milled and sanded surfaces, the shear strength of the joint increased statistically significantly after UV radiation of 2.36 J/cm^2^, but only after 45.2 J/cm^2^ for joints with planed surfaces. There was an increase in average shear strength of 27% in sanded joints, 42% in face-milled joints, and 22% in planed joints, compared with the control samples. A sharp increase in shear strength was therefore the result of the glue’s better mechanical and chemical adhesion. This is because sanding and face milling lead to fibrillation and proper longitudinal cutting of the cells; cells are exposed on the surface; UV light is allowed to reach the pits and the middle lamella very intensively. In contrast, light cannot reach the central lamella so intensely on planed (plasticised) surfaces, where cells are crushed rather than cut. When hydrophobic lignin degrades, surface hydrophilicity is increased because cellulose becomes more abundant on the wood surface. More surface area is available for mechanical anchorage with an adhesive, potentially contributing to improved adhesion strength. Moreover, UV light also causes pits to open, allowing coatings and adhesives to penetrate deeper into the wood, enhancing mechanical anchoring.

However, after the sharp increase in shear strength of face-milled and sanded joints with short-term intense UV irradiation, the trend did not follow the same pattern. In both cases, the shear strength was initially relatively constant, and then, at higher doses of irradiation, it increased in the sanded joints, while it decreased in the face-milled joints. The relationship between wetting and shear strength at high doses of radiation is connected with the proportion of adhesion failure, rapid drying of glue and wood surfaces, and surface morphology after machining.

Furthermore, several procedures must be carried out to identify all the key parameters more precisely to develop this method in greater detail. Short-term UV radiation can suddenly and intensively activate wooden surfaces, so it is necessary to define radiation regimes within this range, taking into account the change in surface energy. Additionally, it is important to solve the technological problem of drying the glue, for example, by extending the open time of the glue, in order to achieve a complete joining of the joints and to determine how this impacts the strength of the joint. Depending on the results, further research would be conducted with other wood types and the possibilities of technological applications to improve the strength of glued joints, as well as the adhesion and durability of wood coatings.

## Figures and Tables

**Table 2:** Characteristics of the medium-pressure Hg-lamp (Heraeus) used.

Medium-Pressure Hg-Lamp Properties	
Effective Spectral Range (nm)	200–300
Specific Electrical Power (W/cm)	80–120
Specific Radiation Flux UVC (W/cm)	12–18
Power Range (kW)	0.5–15
Surface Temperature (°C)	600–900

**Table 3:** Irradiation levels.

Irradiation Level (Number of Passes)	0 (Control)	1	5	10	20	50
Dose (J/cm^2^)	0	2.36	11.80	23.60	45.20	118.00

**Table 4:** Selected bonding parameters and adhesive properties for PVAc adhesive.

Bonding Parameters and Adhesive Properties	
Base	PVAc dispersion
pH value	3 ± 0.5
Viscosity at 20 °C (mPas)	12,000 ± 3000
Open time at 20 °C (min)	6–10
Water Resistance (EN 204)	Stress Group D3

## Data Availability

The data presented in this study are available upon request from the corresponding author.
